# Determination of level of self‐reported adherence of antihypertensive drug(s) and its associated factors among patient with hypertension at a tertiary care center

**DOI:** 10.1111/jch.14592

**Published:** 2022-10-27

**Authors:** Laxman Bhusal, Bishnu Deep Pathak, Bishal Dhakal, Nabin Simkhada, Neeraj Sharma, Binit Upadhaya Remi, Sushil Adhikari, Prakash Raj Oli, Shashank Neupane, Binod Limbu, Dhan Bahadur Shrestha

**Affiliations:** ^1^ Institute of Medicine Maharajgunj Nepal; ^2^ Nepalese Army Institute of Health and Sciences Nepal; ^3^ Jibjibe Primary Health Care Centre Rasuwa Nepal; ^4^ Karnali Care International Hospital and Research Center Pvt. Ltd. Surkhet Nepal; ^5^ Mount Sinai Hospital Chicago Illinois USA

**Keywords:** anti‐hypertensive, compliance, hypertension, medication adherence

## Abstract

The study aimed to determine the level of self‐reported adherence to antihypertensive drug(s) and its associated factors among patient with hypertension at a tertiary care center. The authors performed hospital based observational cross‐sectional study using semi‐structured questionnaires, WHO STEP tool and Hill and Bone high blood pressure compliance scale from December 1, 2021 to February 28, 2022. Descriptive statistics, Chi‐square/Fisher's exact test and non‐parametric tests were used for statistical analysis. Among 150 cases included in the study, majority (94, 62.67%) had good adherence based on Hill and Bone high blood pressure compliance scale with adherence level labelled as “good adherence” (score 3) and “not good adherence” (score < 3). The adherence to drug therapy was significantly better in females compared to males (50 [71.43%] vs. 44 [55.00%], *p* = .038). Among the factors related to hypertension and anti‐hypertensive therapy, people with higher body mass index (ρ = ‐.324, *n* = 56, *p* = .015) and taking three or more pills (6, 1.71%, *p* = .017) had lower adherence to therapy. Likewise, forgetfulness (30, 53.57%), ineffective counseling (7, 12.50%), and missed follow‐up (13, 23.21%) were the factors associated with lower adherence to anti‐hypertensive therapy. This study finds good adherence among the patients taking anti‐hypertensive medications. However, with the improved education, lesser number of pills and physical fitness help to adhere with the anti‐hypertensive therapy.

## 1 INTRODUCTION

Hypertension is the commonest cardiovascular disease in adults worldwide.^1^ It is a preventable risk factor for premature death and disability.^1^, ^2^ It leads to approximately 9.4 million deaths per year globally.^3^ According to World Health Organization (WHO), hypertension is defined as the sustained elevation of systolic blood pressure ≥ 140 mm Hg and/or diastolic blood pressure ≥ 90 mm Hg on two different occasions.^4^ However, American Heart Association/American College of Cardiology (AHA/ACC) has suggested a new cutoff value of 130/80 mm Hg for the diagnosis of hypertension.^4^ The commonly used antihypertensive drugs include angiotensin‐converting enzyme (ACE) inhibitors, angiotensin receptor blockers (ARB), calcium channel blockers, diuretics, and beta blockers.^1^

Blood pressure is recognized as a universal premorbid factor for many chronic diseases.^1^ One of the main obstacles in the management of hypertensive patients is poor adherence to drug therapy.^5^ treatment adherence is defined as the extent to which the patients’ therapeutic drug intake aligns with the prescribed treatment. In other words, it refers to the process of taking physicians’ prescribed medications more than 80% of the time.^6^ There are myriad of factors that may affect adherence to antihypertensive therapy like age, sex, residence, education status, health literacy, health insurance, anxiety/depression, sleep disturbances, duration of therapy, and drug class.^2^, ^6^, ^7^

The prevalence of hypertension in Nepal is high, but its awareness and control were found to be low.^8^ Poor medication adherence may result in uncontrolled and refractory hypertension that ultimately leads to poor clinical outcomes.^3^, ^10^ Therefore, the study of drug adherence in hypertensive patients is of vital importance. These types of studies are being conducted very rarely in our setting. The main objective of this study was to determine the level of adherence to antihypertensive treatment in a tertiary care setting, and the various factors associated with it.

## 2 METHODS

### 2.1 Study setting

The study was conducted in the medical out‐patient department (OPD) of a tertiary care center in Kathmandu, Nepal. It is a teaching hospital with 635 beds in total.

### 2.2 Study design and participants

This study was a single‐center, prospective, analytical, cross‐sectional study conducted in medical OPD of a tertiary care hospital. The cases who were already diagnosed with hypertension under medication by the consultant physician were taken for our study.

### 2.3 Sampling and sample size

Non‐probability consecutive sampling method was used. All the patients diagnosed with hypertension under medication were taken for the study. The minimum sample size was calculated by using Cochran's formula as 145.02; with a prevalence of 24.5%,^9^ a confidence interval of 95%, and a margin of error of 7%. However, considering the non‐response rate of 3%, the final sample size was approximately 150.

### 2.4 Data collection and study variables

The required data for the study was collected using semi‐structured questionnaires from interviews and patients’ treatment books. Data collection was done for 3 months, starting from December 1, 2021, and continuing until February 28, 2022.

The data collected using semi‐structured proforma were the patient's demographic information including age, sex, caste, educational status, residence, and occupational status. The behavioral factors included were smoking and alcohol intake, exercise per week, and high salt intake in the diet. The disease‐related factors were time since diagnosis of hypertension (duration of hypertension), group of anti‐hypertensives drugs taken, a number of antihypertensive pills per day, family history of hypertension, frequency of measurement of blood pressure, any emergency hospital visit for hypertensive crisis, and comorbidities. The anthropometric data like height and weight were collected using the WHO STEP tool. The body mass index (BMI) was then calculated with the available data from the STEP questionnaire.^12^

To determine the level of adherence to antihypertensive medication, we used a three questionnaires model that was adapted from Hill and Bone high blood pressure (HBP) compliance scale.^10^ It was an easy and simple measure to measure the adherence level in hypertensive patients. The three model questionnaires adopted were as follows:

1. Do you ever forget to take your high BP medication?
2. Do you ever miss taking your high BP pills when you feel sick?
3. Do you ever miss taking your high BP pills when you feel better?

The answers for each question were labeled as “Yes” or “No” and 1 score was given for each “No” answer, and a score of 0 was given for the “yes” answer. The possible maximum and minimum scores were 3 and 0, respectively. A score of 3 was taken as good adherence, and less than 3 was taken as “not good adherence.”

The possible factors affecting non‐adherence to the anti‐hypertensive medications were speculated based on the previous research on adherence to HBP medication and interviews with the patients.

### 2.5 Ethical consideration

The ethical approval was taken from Institutional Review Committee (IRC Reg. No. 440), Nepalese Army Institute of Health Sciences. For the conduction of the study, permission was taken from the hospital authority and the head of the department (HoD). The informed verbal consent was taken from the patients. The privacy and anonymity of patient information were well‐maintained. The formal research protocol registration was obtained from the institutional review committee of Nepalese Army Institute of Health Sciences (IRC Reg. No. 440). The STROCSS checklist was followed in reporting the findings and writing the manuscript.^25^

### 2.6 Data analysis

The statistical analysis was run by using Statistical Package for the Social Sciences (IBM‐SPSS), version‐23. The dependent variable was the level of adherence. And, all the other variables affecting adherence were independent. The histogram and Shapiro‐Wilk W test were performed to check the normality of continuous data. The Median/interquartile range (IQR) was calculated for continuous variables. Chi‐square/Fisher's exact test was applied to check the association between independent and dependent variables among categorical variables. Frequency and percentages were presented appropriately in tables. As continuous variables were not normally distributed, Mann‐Whitney U test/Kruskal‐Wallis H test was applied based on the number of variables to check the association with the dependent variable.

A sub‐group analysis was performed separately among those who had “no good adherence” to assess the possible factors affecting their adherence to drug therapy. The significance level was taken as *p* < .07, with a 95% confidence interval and a margin of error of 7%.

## 3 RESULTS

A total of 150 cases diagnosed with hypertension were taken and analyzed. Among them, the majority (94, 62.67%) had good adherence and 56 (37.33%) did not have good adherence. The total median adherence score was 3.00 (2.00–3.00), with minimum and maximum values being 0 and 3, respectively.

The total cases were divided into two groups: “good adherence” (score 3) and “not good adherence” (score < 3). The median score in the latter group was 2.00 (1.00–2.00). The comparison was done between these two groups based on different factors as shown in Table 1.

**TABLE 1:** Socio‐demographic and clinical characteristics

**SN**	**Variables**	**Total**	**Good adherence**	**Not good adherence**	***p*‐value**
1	Age category Less than 40 41–50 51–60 More than 60	9 (100.00) 16 (100.00) 46 (100.00) 79 (100.00)	5 (55.56) 9 (56.25) 35 (76.09) 45 (56.96)	4 (44.44) 7 (43.75) 11 (23.91) 34 (43.04)	.163
2	Sex Male Female	80 (100.00) 70 (100.00)	44 (55.00) 50 (71.43)	36 (45.00) 20 (28.57)	**.038**
3	Caste Brahmin Chhetri Newar Others	25 (100.00) 67 (100.00) 14 (100.00) 44 (100.00)	15 (60.00) 42 (62.69) 8 (57.14) 29 (65.91)	10 (40.00) 25 (37.31) 6 (42.86) 15 (34.09)	.928
4	Residence Urban Rural	80 (100.00) 70 (100.00)	55 (68.75) 39 (55.71)	25 (31.25) 31 (44.29)	.100
5	Marital Status Married Widower	144 (100.00) 6 (100.00)	91 (63.19) 3 (50.00)	53 (36.81) 3 (50.00)	.672
17	Education status Illiterate Read and write Primary Secondary Above secondary	59 (100.00) 29 (100.00) 26 (100.00) 29 (100.00) 7 (100.00)	38 (64.41) 17 (58.62) 14 (53.85) 19 (65.52) 6 (85.71)	21 (35.59) 12 (41.38) 12 (46.15) 10 (34.48) 1 (14.29)	.586
18	Employment status Not working Working	21 (100.00) 129 (100.00)	13 (61.90) 81 (62.79)	8 (38.10) 48 (37.21)	.938
6	Physical exercise per week Never 1–2 times Everyday	73 (100.00) 36 (100.00) 41 (100.00)	44 (60.27) 20 (55.56) 30 (73.17)	29 (39.73) 16 (44.44) 11 (26.83)	.236
7	High salt intake Yes No	53 (100.00) 97 (100.00)	32 (60.38) 62 (63.92)	21 (39.62) 35 (36.08)	.668
8	Smoking habit Never smoked No longer smoker Current smoker	64 (100.00) 69 (100.00) 17 (100.00)	43 (67.19) 40 (57.97) 11 (64.71)	21 (32.81) 29 (42.03) 6 (35.29)	.538
9	Alcohol intake Never Social Reformed Regular	84 (100.00) 30 (100.00) 33 (100.00) 3 (100.00)	56 (66.67) 17 (56.67) 20 (60.61) 1 (33.33)	28 (33.33) 13 (43.33) 13 (39.39) 2 (66.67)	.519
10	Duration of hypertension Less than 1 year More than 1 year	10 (100.00) 140 (100.00)	7 (70.00) 87 (62.14)	3 (30.00) 53 (37.86)	.744
11	Number of anti‐hypertensive pills per day One Two Three or more	89 (100.00) 47 (100.00) 14 (100.00)	54 (60.67) 32 (68.09) 8 (57.14)	35 (39.33) 15 (31.91) 6 (42.86)	.630
12	Group of anti‐hypertensive ACE inhibitors ARBs CCBs Beta‐blockers Combination of above Others	3 (100.00) 32 (100.00) 43 (100.00) 1 (100.00) 68 (100.00) 3 (100.00)	1 (33.33) 19 (59.38) 23 (53.49) 1 (100.00) 49 (72.06) 1 (33.33)	2 (66.67) 13 (40.63) 20 (46.51) 0 19 (27.94) 2 (66.67)	.630
13	Family history of HTN Present Absent	55 (100.00) 95 (100.00)	37 (67.27) 57 (60.00)	18 (32.73) 38 (40.00)	.375
14	Frequency of BP measurement Everyday Weekly Monthly When available During hospital visit	1 (100.00) 38 (100.00) 21 (100.00) 38 (100.00) 52 (100.00)	1 (100.00) 26 (68.42) 14 (66.67) 24 (63.16) 29 (55.77)	0 12 (31.58) 7 (33.33) 14 (36.84) 23 (44.23)	.674
15	History of HTN urgency or emergency Yes No	34 (22.67) 116 (77.33)	21 (61.76) 73 (62.93)	13 (38.24) 43 (37.07)	.902
16	Co‐morbidities Yes No	101 (67.33) 49 (32.67)	61 (60.40) 33 (67.35)	40 (39.60) 16 (39.60)	.409

### 3.1 Socio‐demographic and baseline clinical characteristics

Out of total cases, 80 (53.33%) were males and 70 (46.67%) were females. The adherence to drug therapy was significantly better in females compared to males (50 [71.43%] vs. 44 [55.00%], *p* = .038). The majority of the cases (79, 52.67%) belonged to the age group of more than 60 years. However, good adherence was most common among aged 51–60 years (35, 76.09%), followed by the above 60 years age group (45, 56.96%). But this was not statistically significant (Table 1 **)**.

The patients living in urban areas had better adherence than those in rural areas (55 [68.75%] vs. 39 [55.71%], *p* = .100). Likewise, good adherence was found maximally in those having above secondary education (6, 85.71%), compared to secondary (19, 65.52%), primary (14, 53.85%), able to read and write (17, 58.62%) and illiterate groups (38, 64.41%). But, none of these were statistically significant. In the same way, there was no significant difference in adherence to drug intake with respect to caste, marital status, and employment status (Table 1 **)**.

Good adherence was found to be highest among those who had been doing some forms of physical exercise every day (30, 73.17%). The assessment was also done based on salt intake in daily meals. High salt intake was defined as the practice of taking added salt to the diet. Those who used to have a high salt diet had lower drug adherence compared to low salt consumers (32 [60.38%] vs. 62 [63.92]). Likewise, adherence was best among those who had never smoked (43, 67.19%), and never consumed alcohol (56, 66.67%). However, these differences were not significant statistically (*p* < .07) (Table 1 **)**.

### 3.2 Factors related to hypertension

The adherence to drug therapy was assessed with regard to the duration of hypertension. It was found to be higher in newly diagnosed cases (less than 1 year) than in old cases (more than 1 year) (7 [70.00%] vs. 87 [62.14]). But, this was not statistically significant. Likewise, there were no significant differences across the group of anti‐hypertensives and the number of pills taken per day. The drug adherence was numerically higher in those, who had a positive family history of hypertension (37, 67.27%). Those patients who used to monitor their blood pressure (BP) weekly (26, 68.42%) or monthly (14, 66.67%) were found to have better adherence, compared to those who got their BP measured only when available (24, 63.16%) or during hospital visit (29, 55.77%). But, this was not significant statistically. Similarly, the patients with co‐morbidities (61, 60.40%) had poorer adherence in comparison to those without any co‐morbidities (33, 67.35%), though this was not statistically significant (*p* = .409) (Table 1 **)**.

### 3.3 Sub‐group analysis

The patients with poor drug adherence were analyzed separately for factors that had significant effects on their drug intake. (Table 2) The total adherence score was mildly negatively correlated with body mass index (BMI). The higher the BMI, the lower was the adherence to therapy (ρ = ‐.324, *n* = 56, *p* = .015).

**TABLE 2:** Sub‐group analysis

**SN**	**Variables**	**Adherence score**	***p*‐value**
1	Number of pills per day		**.017**
	One (35, 62.50%)	2.00 (0–2.00)	
	Two (15, 26.79%)	2.00 (2.00–2.00)	
	Three or more (6, 10.71%)	1.00 (0–1.25)	
2	Forgetfulness		**.001**
	Yes (30, 53.57%)	2.00 (2.00–2.00)	
	No (26, 46.43%)	1.00 (0–2.00)	
3	Ineffective counseling		**.026**
	Yes (7, 12.50%)	1.00 (0–2.00)	
	No (49, 87.50%)	2.00 (1.00–2.00)	
4	Missed to follow‐up		**.008**
	Yes (13, 23.21%)	1.00 (0–2.00)	
	No (43, 76.79%)	2.00 (2.00–2.00)	

Similarly, the median adherence score was significantly higher (*p* = .017) in patients taking one (2.00 [0–2.00]) or two pills (2.00 [2.00–2.00]) per day, compared to those taking three or more pills (1.00 [0–1.25]). This concludes that drug prescription with a lesser number of pills increased the adherence to therapy.

The patients were asked about the factors affecting their adherence to drug therapy. The most common factors mentioned by them were forgetfulness (30, 53.57%), ineffective counseling (7, 12.50%), and missed follow‐up (13, 23.21%). On calculating their adherence score, the median score was significantly lower (*p* = .026) in those, who said “yes” to ineffective counseling (1.00 [0–2.00]), compared to those who denied this cause (2.00 [1.00–2.00]). Similarly, those who admitted “missed follow‐up” as the affecting factor, had a significantly lower (*p* = .008) median score in comparison to those who said “no” to this cause (1.00 [0–2.00] vs. 2.00 [2.00–2.00]). Thus, the two factors “ineffective counseling” and “missed follow‐up” were found to have significantly affected their adherence to drug therapy.

## 4 DISCUSSION

In our study, the majority of the patients (62.67%) had good adherence (adherence score of 3) to anti‐hypertensive treatment. The drug adherence in the rest of the patients (37.33%) was poor (score < 3). In contrast, a study in Eastern Nepal depicted that 56.5% of patients were adherent to antihypertensive therapy.^11^ However, the adherence level was reported comparatively lower (35.4%) in another study in Nepal by Khan and coworkers^12^ Likewise, most of the participants (61.1%) in research done in Pakistan were found to be non‐adherent.^13^ Another study conducted in an African country depicted that 64.4% of patients were poorly adherent to medication, and it was significantly associated with uncontrolled blood pressure.^14^ In this way, drug adherence in our setting is found to be better than in most other countries across the globe. This discrepancy could be due to different study populations and sample sizes. Moreover, different scales were used for measuring drug adherence in these studies.

In the present study, drug adherence was significantly better in females (50, 71.43%) compared to males (39, 55.71%). A similar finding was also reported in a study by Pan and coworkers^3^ But, this is in contrast to another study which showed greater adherence in males compared to females.^2^ These differences could be due to differences in the study population and sample size. The age of patients also influences their adherence to therapy. A study by Lor M depicted that drug adherence scores decreased with an increase in age.^2^ One explanation for this finding could be that deterioration of cognitive status with aging may lead to poor adherence to treatment.^15^, ^16^, ^17^ However, there are a few other studies where compliance to therapy was better in patients with older age compared to younger ones.^18^, ^19^, ^20^ In our study, good adherence was common among the age group of 51–60 years and above 60 years, even though it was not statistically significant.

Similarly, the education level of patients also significantly affects drug adherence. The studies by Lor and coworkers^2^ and Kripalini and coworkers^20^ concluded that adequate health literacy was associated with higher adherence to treatment. In our case, patients with above secondary level education had greater adherence to therapy compared to others. But it was not statistically significant. Despite these differences, education and support to patients help to improve their adherence level.^6^

Residence also influences compliance with treatment. A study on the Chinese population showed that drug adherence was greater in residents of urban areas compared to those of rural places.^3^ Similar finding was also reported in our study even though it is not statistically significant.

Many other factors can influence drug adherence in patients with hypertension. These include anxiety, depression, sleep disturbance, and family income.^2^ The duration of hypertension also plays an important role. The longer the patient is diagnosed with high blood pressure, the more adherent s/he is to the treatment.^1^ However, our study could not illustrate any significant association between drug adherence and the duration of hypertension. Likewise, the factors responsible for non‐adherence as reported in a study in Nepal were illiteracy, the expensive price of medicine, missed medicine due to cost, no family history of hypertension, irregular follow‐up, and multiple pills per day.^21^

There can be several factors affecting adherence to anti‐hypertensive therapy as described above. However, based on our sub‐group analysis, adherence was poor in people taking more than two anti‐hypertensive pills. Similarly, those who missed to follow‐up on a regular basis and who did not get adequate counselling about the use of anti‐hypertensive medications were among the ones with poor adherence with anti‐hypertensive therapy. These finding were consistent with the previous studies.^6^, ^11^, ^12^, ^21^

There are a few limitations of our study. It's a single‐center cross‐sectional study with a smaller sample size. Non‐probability consecutive sampling was adopted. So, all the findings may not be generalizable to a larger population or whole country.

Adherence to antihypertensive treatment is found to have improved blood pressure, decreased hospitalization rate, and lower medical care costs.^22^ Non‐adherence to therapy poses a major risk for cardiovascular complications.^6^, ^23^ The clinicians need to explore the possible causes of sub‐optimal adherence or non‐adherence to the treatment of hypertension.^24^ Education and counseling to the patients, motivation, and support, simplification of pharmacological regimens, and formulation of health programs to assist the patients are a few intervention to improve drug adherence level.^6^, ^13^

## 5 CONCLUSIONS

The medication adherence to anti‐hypertensive therapy, as found in our study, was good. However, there are still many loop holes behind non‐adherence to the therapy which are of primary concern to the treating physicians. Strategies like lower number of pills, improved education level and BMI within the reference range can help in adherence with the anti‐hypertensive medications.

## AUTHORS CONTRIBUTION

Laxman Bhusal: Contributed in proposal making, supervision and literature review, Bishnu Deep Pathak: Contributed in literature review and writing manuscript, Bishal Dhakal: Contributed in literature review, data collection, writing and editing manuscript, Nabin Simkhada: Contributed in manuscript review and editing, Neeraj Sharma: Contributed in supervision and literature review, Binit Upadhaya Regmi: Contributed to manuscript review and editing, Sushil Adhikari: Contributed in data entry and collection, Prakash Raj Oli: Contributed in manuscript editing and supervision, Shashank Neupane: Contributed in manuscript review and editing, Binod Limbu: Contributed in data collection and literature review, Dhan Bahadur Shrestha. Contributed in manuscript review and editing. All the authors read and approved the final manuscript.

## CONFLICT OF INTEREST

All authors declare no conflict of interest.
